# The Molecular Sieving of Propylene and Propane on SAPO-35 Molecular Sieve

**DOI:** 10.3390/nano15231820

**Published:** 2025-12-01

**Authors:** Yansi Tong, Kadi Hu, Qihao Yang, Hao Liu, Danhua Yuan, Jungang Wang, Mengting Lv, Hailong Wang, Ziqi Tian, Yunpeng Xu, Liang Chen

**Affiliations:** 1 Zhejiang Key Laboratory of Advanced Fuel Cells and Electrolyzers Technology, Ningbo Institute of Materials Technology and Engineering, Chinese Academy of Sciences, Ningbo 315201, China; 2 University of Chinese Academy of Sciences, Beijing 100049, China; 3 Dalian National Laboratory for Clean Energy, Dalian Institute of Chemical Physics, Chinese Academy of Sciences, Dalian 116023, China; 4 China State Key Laboratory of Coal Conversion, Institute of Coal Chemistry, Chinese Academy of Sciences, Taiyuan 030001, China

**Keywords:** propylene/propane separation, silicoaluminophosphate molecular sieve, pore engineering, molecular sieving

## Abstract

Selective adsorption is regarded as a promising alternative for propylene/propane separation. However, the similar physicochemical properties of these two components pose a challenge in developing adsorbents that simultaneously exhibit high selectivity and substantial adsorption capacity. This study aims to achieve molecular sieving of propylene and propane by precisely controlling the pore size of silicoaluminophosphate (SAPO) molecular sieve. The pore size of the eight-membered-ring SAPO-35 molecular sieve is tuned via ion exchange to fall between the kinetic diameters of propylene and propane, enabling selective adsorption of propylene while excluding propane molecules. Ion exchange treatment increased the equilibrium adsorption selectivity of the SAPO-35 from 2.2 to 11.4, placing it among the highest-performing molecular sieve-based adsorbents. This modification also substantially improved the material’s regeneration capability at ambient temperature. Theoretical calculations reveal that steric hindrance effects, arising when gas molecules diffuse through the eight-membered-ring channels, contribute significantly to the high adsorption selectivity. Breakthrough experiments demonstrated that Na-SAPO-35 achieves a dynamic selectivity of 15.9 for propylene/propane separation. The development of Na-SAPO-35 adsorbents with high selectivity, substantial adsorption capacity, and robust durability is critical for advancing the industrial implementation of adsorption-based separation technologies.

## 1. Introduction

The combustion of fossil fuels releases substantial quantities of carbon dioxide (CO_2_), which has precipitated climate change and ecological shifts, emerging as critical challenges confronting contemporary society [1]. To mitigate this challenge, significant research efforts have been directed toward the catalytic conversion and utilization of CO_2_. The use of CO_2_ as an oxidant in propane dehydrogenation (PDH) simultaneously reduces carbon emissions, lowers reaction temperature, mitigates carbon deposition, and suppresses excessive oxidation [2,3]. CO_2_-PDH is an efficient propylene production technology with greater industrial application potential. However, obtaining high-purity propylene from the product stream is complicated by the difficulty in separating propane, owing to their nearly identical molecular weights, boiling points, and polarities [4,5]. Pressure swing adsorption (PSA)—a process involving cyclic selective adsorption and desorption of gases on adsorbents—is considered a viable alternative to energy-intensive cryogenic distillation, offering reduced operational costs and capital expenditure [6,7]. The performance of PSA in competing with cryogenic distillation hinges on adsorbents exhibiting high selectivity, substantial capacity, rapid adsorption–desorption kinetics, and long-term durability [8].

Adsorptive separation of propylene/propane mixtures can be performed under static or dynamic conditions. The former mode relies on the difference in equilibrium adsorption capacities between propylene and propane, which stems from their distinct interactions with the adsorbent [9]. In contrast, the latter mode exploits difference in diffusivity between the two components [10]. When the pore size of the adsorbent lies between the molecular dimensions of propylene and propane, propylene is selectively adsorbed while propane is completely excluded—a process termed molecular sieving, which offers the highest selectivity [11]. However, achieving molecular sieving for propylene and propane is exceptionally challenging due to their similar molecular sizes (4.68 Å for propylene and 4.3–5.12 Å for propane) [10]. Eight-membered-ring molecular sieves are promising candidates because of their uniform channels, robust stability, and pore sizes comparable to the molecular dimensions of the target gases [12]. For instance, aluminosilicate 4A zeolite has been used for propylene/propane separation, achieving adsorption capacities of 1.9 mmol g^−1^ for propylene and 0.2 mmol g^−1^ for propane [13,14]. However, approximately 10% of propylene remains trapped without heat treatment, and carbon deposition during regeneration can block the adsorbent’s micropores [15]. Other materials like all-silica zeolites (e.g., DD3R [16], Si-CHA [17]) and aluminophosphate molecular sieves (e.g., AlPO-14 [15]) also exhibit excellent molecular sieving performance. Yet, their complex synthesis processes and high costs hinder widespread application.

Silicoaluminophosphate molecular sieves with diverse topology structures are widely used in catalytic synthesis but are rarely reported for adsorption application [18,19]. Among them, the eight-membered-ring SAPO-17 (ERI, 3.6 Å × 5.1 Å) has been applied in the kinetic separation of propylene and propane, achieving a kinetic selectivity of 1980 and demonstrating excellent reproducibility [20]. Additionally, when the pore aperture of the molecular sieve is reduced to a size between that of propylene and propane molecules, the resulting adsorbent can selectively adsorb propylene while effectively excluding the larger propane molecules due to steric hindrance.

In this study, the silicoaluminophosphate molecular sieve SAPO-35 (LEV, 3.6 Å × 4.8 Å) was synthesized via a hydrothermal method, and its pore size was further tuned through ion-exchange treatment. The resulting sodium-form SAPO-35 (Na-SAPO-35) exhibited an equilibrium adsorption selectivity of up to 11.4, with negligible loss in propylene adsorption capacity over three consecutive regeneration cycles. Breakthrough experiments demonstrated a dynamic adsorption selectivity of 15.9 for the separation of propylene and propane. Theoretical calculations revealed that the molecular sieving effect stems from the steric hindrance encountered by propane molecules during diffusion through the eight-membered-ring pores. This study achieved precise control over the pore size of the silicoaluminophosphate molecular sieve via ion exchange and successfully applied it to propylene/propane molecular sieving, thereby expanding the range of viable adsorbent materials.

## 2. Materials and Methods

### 2.1. Materials and Reagents

Hexamethyleneimine (C_6_H_13_N, 98%), sodium chloride (NaCl, 99.5%), and ammonium chloride (NH_4_Cl, 99%) were purchased from the Aladdin Chemical Reagent Co., Shanghai, China. Phosphonic acid (H_3_PO_4_, 85%) was purchased from Damao Reagent Co., Tianjin, China. Pseudo-boehmite (Al_2_O_3_, 67.5%) was purchased from Shandong Aluminum Industry Co., Zibo, China. Silica sol (SiO_2_, 27.3%) was purchased from Shenyang Chemical Industry Co., Shenyang, China. All of the above reagents were used without further purification.

### 2.2. Synthesis and Modification of SAPO-35

The gel preparation was performed in a 90 °C water bath. Phosphoric acid (4.43 g) and pseudo-boehmite (3.09 g) were added to deionized water (20 g) under continuous magnetic stirring. Stir thoroughly for 3 h to obtain a white emulsion. Subsequently, silica sol (0.44 g) was added dropwise, followed by stirring for 1 h. Finally, template hexamethyleneimine (2.60 g) was added dropwise, and the mixture was immediately transferred to a stainless-steel autoclave. The reaction gel composition was 1 Al_2_O_3_:0.96 P_2_O_5_:0.1 SiO_2_:1.31 HMI:55 H_2_O. Crystallization proceeded at 200 °C for 24 h in a rotating oven. After cooling to room temperature, filtered, washed with deionized water, and dried to yield a solid product. The synthesized solid was calcined in a muffle furnace at 700 °C for 6 h to remove the organic template, yielding hydrogen-form SAPO-35 (H-SAPO-35).

H-SAPO-35 (2 g) was added to 1 mol L^−1^ NH_4_Cl solution (100 mL) and stirred thoroughly at 60 °C for 3 h. The solid was filtered and washed with deionized water until no Cl^−^ ions were detected, yielding ammonium-form SAPO-35 (NH_4_-SAPO-35) molecular sieve. The obtained NH_4_-SAPO-35 was added to NaCl solution (100 mL) with a liquid-solid mass ratio of 50:1. The mixture was stirred thoroughly at 60 °C for 1 h. The solid was filtered and washed with deionized water until no Cl^−^ ions were detected. The Na^+^ ion exchange process was repeated twice to obtain Na-SAPO-35.

### 2.3. Breakthrough Experiments

The adsorbents (40–60 mesh) were packed into a stainless-steel tube (0.3 m in length, 8 mm in inner diameter). The sample underwent pretreatment at 350 °C for 4 h under a helium purge flow of 10 mL min^−1^. Breakthrough experiments were performed at room temperature. The feed gas (C_3_H_6_:C_3_H_8_ = 50:50, *v*/*v*) was introduced into the bed, and the composition of outlet gas was analyzed by mass spectrometry. The adsorption amount of a component in the mixture during the breakthrough experiment (*q_i_*) was calculated as follows:

$$q_{i}=\frac{F\left(C_{i,0}t_{b}-\int_{0}^{t_{b}}C\left(t\right)d_{t}\right)-V_{d}}{m_{a}}$$

where *F* (mL min^−1^) is the flow rate of feed gas, *C_i_*_,0_ is the concentration fraction of component *i* in the feed gas, *C_t_* is the concentration fraction of component *i* in exit gas, *t_b_* (min) is the breakthrough time of component *i*, *V_d_* (mL) is the dead volume of the adsorption bed, and *m_a_* (g) is the mass of adsorbent.

### 2.4. Theoretical Calculation Method

To account for the substitution in the zeolite framework, the unit cell of the substituted structure was constructed from the crystallographic information file (CIF) obtained from the database and subsequently relaxed using density functional theory (DFT) geometry optimization. The optimized lattice parameters are a = b = 13.30 Å, c = 21.90 Å, α = β = 90°, and γ = 120°. A 10 × 10 × 10 supercell was then constructed and used for grand canonical Monte Carlo (GCMC) simulations. The gas molecules, i.e., C_3_H_6_ and C_3_H_8_, were modeled using the full atomic model, with fixed bond lengths and bond angles (1.34 Å for C=C bond length of propylene, 1.54 Å for C–C bond length of propane). The CVFF potential was used to describe the intermolecular interactions. The grand canonical ensemble (*μVT*) was adopted, with fixed temperature (298 K), volume, and gas chemical potential (μ). Gas molecules were randomly generated within the pores, and the acceptance probability was determined by the Boltzmann factor *P* = min (1, exp[(*μ*–*U*)/kT]) (*U* is the interaction energy between the molecule and the system). The total sampling steps were 1 × 10^7^, with the first 5 × 10^6^ steps being the equilibration period (no statistics were recorded), and the last 5 × 10^6^ steps being the sampling period. The uptake was calculated every 100 steps to ensure data convergence. The equilibrium number of molecules (*N*) obtained from the simulation was converted to molar adsorption amount as *n* in mmol g^−1^. The chemical potential was converted to pressure (*P*) using the ideal gas state equation *μ* = *μ*_0_ + k*T* ln (*P*/*P*_0_). The isotherms were plotted with *P* versus *n*.

### 2.5. Characterizations

Crystalline phase was identified on a powder X-ray diffraction (XRD, PANalytical X’Pert PRO diffractometer, Almelo, The Netherlands) with Cu-Kα radiation (λ = 1.54059 Å) at 40 kV and 40 mA. Chemical compositions were analyzed using an X-ray fluorescence spectrometer (XRF, Philips Magix-601, Amsterdam, The Netherlands). The microscopic morphology was characterized using a field emission scanning electron microscopy (SEM, Hitachi SU8020, Tokyo, Japan). ^1^H NMR spectra was acquired on a solid-state magic-angle spinning spectrometer (Bruker Avance III-600, Karlsruhe, Germany) using a 4 mm probe. The micropore properties were evaluated at 77 K, and the equilibrium adsorption capacity was measured in a 25 °C water bath using a physical adsorption analyzer (Micromeritics ASAP 2020, GA, USA). Thermal analysis was performed using a thermal analyzer (TA SDT 600, DE, USA) with the temperature ramped from room temperature to 700 °C under an air atmosphere.

## 3. Results and Discussion

### 3.1. Characterization of Adsorbents

**Figure 1:**
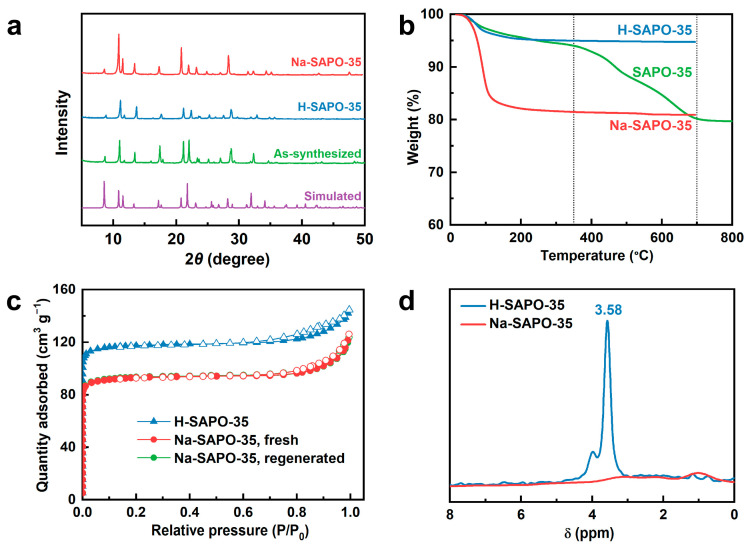
(**a**) XRD patterns of simulated LEV zeolite, as-synthesized sample, H-SAPO-35, and Na-SAPO-35. (**b**) The thermogravimetric curves of samples. (**c**) N_2_ adsorption–desorption isotherms of samples at 77 K (close symbols, adsorption; open symbols, desorption). (**d**) ^1^H MAS NMR spectra of samples.

The X-ray diffraction (XRD) pattern of the as-synthesized molecular sieve exhibits diffraction peaks identical to those of the simulated LEV zeolite (Figure 1a) [21], confirming successful synthesis of SAPO-35 with high crystallinity and purity. After calcination and subsequent ion-exchange treatment, the crystalline structures of H-SAPO-35 and Na-SAPO-35 remained largely unchanged despite the disappearance of some weak peaks, demonstrating good thermal and hydrothermal stability. The micromorphology of H-SAPO-35 and Na-SAPO-35 (Supplementary Materials Figure S1) both show short hexagonal columns with a size of about 20 μm, formed by stacking of Nano-scale lamellar crystals, indicating that the ion-exchange treatment did not damage the crystalline structure. As shown in Figure 1c, the thermogravimetric curve of as-synthesized SAPO-35 shows two weight loss peaks, corresponding to the removal of water and the combustion of the template. The thermogravimetric curves of H-SAPO-35 and Na-SAPO-35 show only a weight loss peak below 250 °C due to dehydration, indicating that the templates within the framework were completely removed during calcination. The low-temperature adsorption isotherms of H-SAPO-35 and Na-SAPO-35 (Figure 1c) are both type I, with a hysteresis loop observed between 0.7 and 1, indicating that both samples are typical microporous materials containing some mesopores [22]. As shown in Supplementary Materials Table S1, compared with H-SAPO-35 (345 m^2^ g^−1^ and 0.21 cm^3^ g^−1^), the micropore surface area and pore volume of Na-SAPO-35 (255 m^2^ g^−1^ and 0.17 cm^3^ g^−1^) decrease after ion-exchange treatment, as the exchanged sodium ions block some micropores, rendering them inaccessible to nitrogen probe molecules [23].

The silicon content in H-SAPO-35 is low (2.8%, Supplementary Materials Table S2), so each silicon atom incorporates into the framework via the SM2 mechanism, generating a Brønsted acid site [24,25], resulting in an equivalent content of Bronsted acid sites and silicon. After ion exchange, the Na^+^ content equals that of the Brønsted acid sites, indicating that the protons in the SAPO-35 framework have been completely replaced by Na^+^ ions [26]. The ^1^H NMR spectrum of H-SAPO-35 (Figure 1d) shows a strong peak at 3.58 ppm, attributed to protons in bridged hydroxyl groups, confirming the presence of Brønsted acid sites in H-SAPO-35 [27,28]. After ion exchange, this peak nearly disappears from the spectrum, revealing almost complete replacement of protons by sodium ions. Therefore, Na-SAPO-35 is Brønsted acid-free, consistent with the compositional analysis. Characterization of physicochemical properties indicates that the eight-membered-ring microporous SAPO-35, free of Brønsted acid sites, has been successfully synthesized.

### 3.2. Equilibrium Adsorption Performance

**Figure 2:**
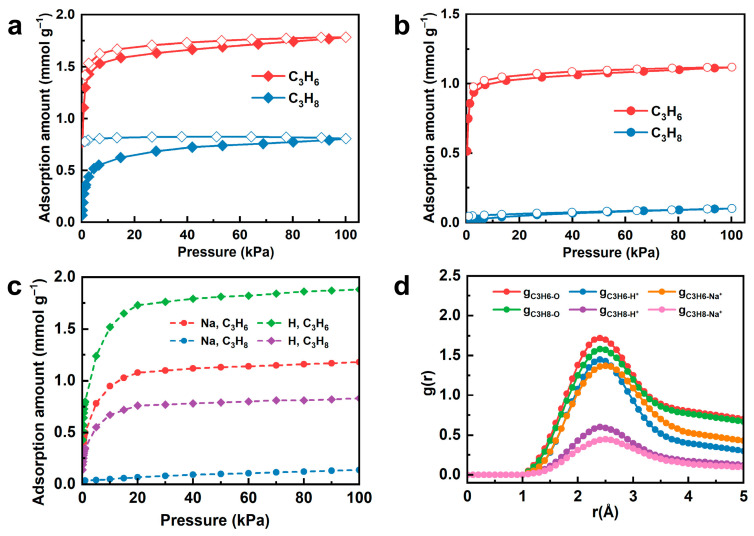
Adsorption–desorption isotherms of propylene and propane on (**a**) H-SAPO-35 and (**b**) Na-SAPO-35 at 298 K (close symbols, adsorption; open symbols, desorption). (**c**) The simulated adsorption isotherms of propylene and propane on H-SAPO-35 and Na-SAPO-35. (**d**) Radial distribution functions of propylene and propane with Na^+^, H^+^ and the oxygen in the frameworks.

The equilibrium adsorption and desorption isotherms of propylene and propane on the adsorbents were measured at 298 K (Figure 2a,b). The adsorption of propylene on H-SAPO-35 increases sharply within 0–5 kPa, indicating a strong affinity between propylene and the adsorbent [29]. Subsequently, it increases steadily with pressure, reaching 1.78 mmol g^−1^ at 100 kPa. In contrast, propane adsorption increases more slowly, reaching 0.81 mmol g^−1^ at 100 kPa. The equilibrium selectivity, defined as the ratio of adsorption capacities, was calculated as 2.2. The open pore structure of H-SAPO-35 permits diffusion and adsorption of both propylene and propane, while the equilibrium adsorption selectivity stems from the stronger thermodynamic interaction between propylene and H-SAPO-35 [30]. The propylene adsorption capacity on Na-SAPO-35 was measured as 1.14 mmol g^−1^ at 100 kPa, lower than that on H-SAPO-35. This decrease is attributed to the reduced micropore surface area and the weakened propylene-adsorbent interaction resulting from Brønsted acid removal [23]. Notably, the adsorption capacity of propane drops sharply to 0.10 mmol g^−1^ at 100 kPa, and the equilibrium selectivity reaches 11.4, ranking among the top tier (Supplementary Materials Table S3). Propane exhibits weak affinity for molecular sieve frameworks due to the absence of unsaturated bonds [31]. We infer that exchanged metal ions tune the eight-membered-ring pore size, thereby blocking diffusion of larger propane molecules via steric hindrance. The dual-site Langmuir model describes these adsorption isotherms with correlation coefficients exceeding 0.998 (Supplementary Materials Table S4), suggesting that the adsorption process involves interactions with two types of adsorption sites possessing different affinities [32].

To elucidate the separation performance of the two adsorbents, grand canonical Monte Carlo (GCMC) simulations were conducted. As shown in Figure 2c, the simulated isotherms agree well with the experimental data. At 100 kPa, the simulated and experimental uptakes of propylene (1.88 vs. 1.78 mmol g^−1^) and propane (0.83 vs. 0.81 mmol g^−1^) for H-SAPO-35 are nearly identical, with deviations of only 5.6% and 2.5% respectively. For Na-SAPO-35, the deviations in propylene and propane uptakes are 3.5% (1.14 vs. 1.18 mmol g^−1^) and 38% (0.10 vs. 0.14 mmol g^−1^), respectively. Due to the low adsorption capacity of propane in Na-SAPO-35, the relative deviation appears significant. To understand the uptake variations on H-SAPO-35 and Na-SAPO-35, the radial distribution functions (RDFs) are plotted in Figure 2d, revealing the adsorption mechanism of SAPO-35 molecular sieve. For H-SAPO-35, the g(r) between propylene carbon atoms and H^+^ exhibits a peak at 2.3–2.4 Å with a value of 1.45, indicating strong π-proton interaction that results in high uptake. In contrast, propane exhibits a g(r) peak value of 0.6, indicating weak interaction with H^+^. For Na-SAPO-35, propylene interacts with Na^+^ via electrostatic forces (g(r) = 1.37 at 2.5 Å), whereas propane exhibits a significantly lower peak with Na^+^ (g(r) = 0.45), consistent with the sharp decline in propane adsorption observed experimentally. Additionally, the RDF peaks for propylene and propane relative to oxygen in the H-SAPO-35 framework are similar (1.72 vs. 1.58), resulting in comparable uptakes and a low selectivity of 2.2. By comparison, in Na-SAPO-35, the propylene peak (1.72) is significantly higher than the propane peak (1.28), and propylene exhibits a broader interaction range, accounting for the high selectivity together. Herein, H-SAPO-35 relies on strong Brønsted acid site–propylene interactions, resulting in limited selectivity, whereas Na-SAPO-35 utilizes ion exchange to tailor the pore environment, enabling efficient propylene/propane separation via a combination of steric hindrance and weak van der Waals interactions. These findings are fully consistent with experimental isotherms, providing direct microscopic evidence for the principle of ion-exchange-regulated molecular sieve separation performance.

By adjusting the pore size through ion-exchange treatment, a sharp decrease in propane adsorption capacity was achieved, enabling SAPO-35 to separate the two components with high selectivity.

### 3.3. Regeneration Performance

**Figure 3:**
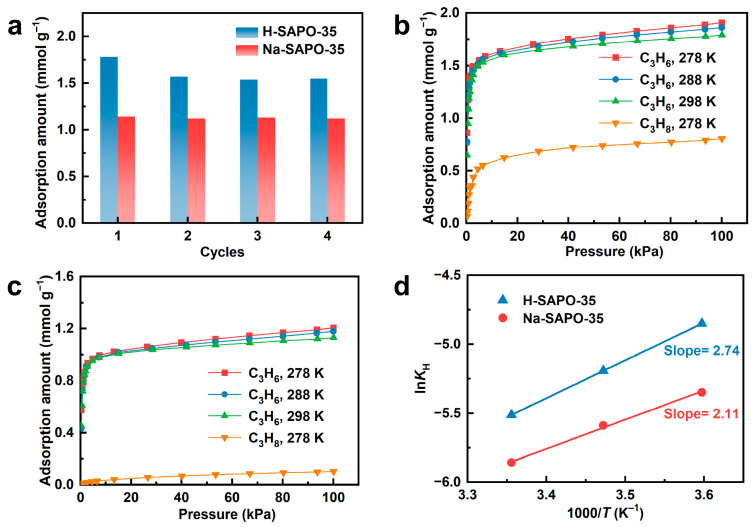
(**a**) Adsorption capacity of propylene on the adsorbents during regeneration experiments. The isotherms of propylene and propane on (**b**) H-SAPO-35 and (**c**) Na-SAPO-35 under different temperatures. (**d**) $lnK_{H}$ − 1/T plots of propylene adsorption on adsorbents.

Beyond adsorption capacity and selectivity, desorption kinetics and reproducibility are crucial for the practical application of adsorbents [33]. Significant hysteresis appears in the propylene adsorption/desorption curves for H-SAPO-35 (Figure 2a), suggesting incomplete desorption during the testing period. Furthermore, the propylene adsorption capacity of H-SAPO-35 decreases to 1.57 mmol g^−1^ during room-temperature regeneration and continues to decline in subsequent cycles (Figure 3a). Protons in Brønsted acid sites interact with propylene unsaturated bonds through π-complex formation, generating carbocation intermediates. These carbocations subsequently react with additional propylene molecules to form oligomers [34,35,36,37], as illustrated in Supplementary Materials Figure S2. The resulting oligomers remain trapped in the channels at room temperature, blocking micropores and reducing propylene adsorption capacity. Moreover, the nearly horizontal propane desorption isotherm for H-SAPO-35 indicates difficult desorption during testing due to slow diffusion kinetics.

The minimal difference between propylene adsorption/desorption curves on Na-SAPO-35 indicates near-complete desorption at room temperature (Figure 2b). Propylene adsorption capacity shows negligible decrease during regeneration (Figure 3a), demonstrating excellent reproducibility. This is confirmed by unchanged micropore surface area and pore volume between regenerated and fresh Na-SAPO-35. Ion-exchange treatment nearly eliminates Brønsted acid sites (Figure 1d), thereby removing the strong interaction between propylene molecules and the molecular sieve framework.

Na-SAPO-35 demonstrates sufficient propylene adsorption capacity as well as negligible propane adsorption, enabling effective molecular sieving of the two gases. Additionally, room-temperature regeneration capability highlights its potential for practical application in PSA-based propylene/propane separation.

### 3.4. Adsorption Thermodynamics

Adsorption thermodynamics studies provide experimental insights into adsorption selectivity. Henry’s law constant (*K*_H_) estimates the adsorbent-adsorbate interaction strength at low concentrations [38,39]. Adsorption isotherms of propylene on H-SAPO-35 and Na-SAPO-35 were measured at 278, 288, and 298 K (Figure 3b,c). Increasing temperature slightly reduces propylene adsorption capacity on both adsorbents. As shown in Supplementary Materials Table S5, the *K*_H_ values (298 K) of propylene and propane on H-SAPO-35 were determined to be 4.04 × 10^−3^ and 5.37 × 10^−4^ mol kg^−1^ Pa^−1^, respectively, resulting in a ratio of 7.5. After ion exchange, *K*_H_ of propylene slightly decreased to 2.86 × 10^−3^ mol kg^−1^ Pa^−1^, whereas the *K*_H_ of propane sharply declined to 1.12 × 10^−5^ mol kg^−1^ Pa^−1^, yielding a greatly enhanced ratio of 255, indicating a significantly stronger adsorption affinity of propylene on Na-SAPO-35. ln*K*_H_−1/T plots (Figure 3d) reveal propylene adsorption heat of 22.8 kJ mol^−1^ on H-SAPO-35 and 17.6 kJ mol^−1^ on Na-SAPO-35, confirming that ion-exchange treatment weakened propylene-framework interaction. These thermodynamic results explain the high equilibrium adsorption selectivity of propylene over propane on Na-SAPO-35 from an experimental perspective.

### 3.5. Breakthrough Experiment

**Figure 4:**
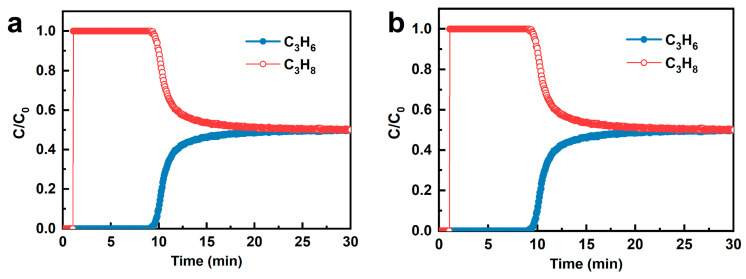
Breakthrough curves of propylene/propane on (**a**) Na-SAPO-35 and (**b**) H-SAPO-35.

The dynamic separation performance of the adsorbents was evaluated using breakthrough experiments. As shown in Figure 4a, propane breaks through the Na-SAPO-35 adsorbent bed at 0.9 min with a dynamic adsorption amount of 0.8 mL g^−1^, identical to its equilibrium adsorption capacity on Na-SAPO-35 (0.8 mL g^−1^, 50 kPa). This suggests near-complete propane exclusion from Na-SAPO-35 during dynamic adsorption. Propylene breakthrough begins at ~7.0 min, and its concentration gradually rises to 50% by 50 min. The total dynamic adsorption amount of propylene is 12.7 mL g^−1^, with a dynamic propylene/propane selectivity (defined as the ratio of dynamic uptakes) of 15.9. The dynamic adsorption amount of propylene on Na-SAPO-35 is lower than its equilibrium adsorption capacity (24.4 mL g^−1^, 50 kPa), due to the slow diffusion kinetics of propylene in the Na-SAPO-35 framework. Consequently, propylene molecules cannot reach inner adsorption sites within the short testing period [40].

H-SAPO-35 breakthrough curves follow a similar trend to Na-SAPO-35. As shown in Figure 4b, the dynamic adsorption capacity of propane (1.0 mL g^−1^) is much lower than its equilibrium capacity (11.2 mL g^−1^, 50 kPa). This discrepancy stems from two factors. First, slow diffusion kinetics hinder propane molecules from reaching internal adsorption sites within the limited contact time [41]. Second, propylene tends to occupy more adsorption sites due to size advantage in the competitive adsorption [42,43]. Propylene mass transfer zone on Na-SAPO-35 (7.0−50.4 min) is significantly longer than that on H-SAPO-35 (9.2−27.9 min), attributed to the slower diffusion kinetics of propylene on Na-SAPO-35, confirming that ion exchange treatment narrows the pore size of the SAPO-35.

## 4. Conclusions

The Brønsted acid sites in eight-membered-ring SAPO molecular sieves can catalyze olefin polymerization even at room temperature, which limits their application in adsorptive separation of light olefins from alkanes. Post-synthetic ion-exchange treatment replaces Brønsted acid site protons, thereby eliminating catalytic activity. Meanwhile, the introduced cations optimize channel dimensions, enabling propylene diffusion while excluding propane. Experimental results show Na-SAPO-35 achieves high equilibrium (11.4) and dynamic (15.9) adsorption selectivity for propylene/propane separation. Additionally, Na-SAPO-35 demonstrates excellent room-temperature regenerability, highlighting its potential for industrial separation processes. The propylene-comparable pore size causes frequent collisions during molecular sieve diffusion, resulting in slower adsorption kinetics and prolonged equilibrium time. In practical PSA applications, this may slightly reduce separation efficiency, necessitating optimization of operating parameters. In summary, the SAPO-35 molecular sieve, with high adsorption selectivity, excellent durability, and low cost, offers a promising alternative for propylene/propane adsorptive separation.

## Data Availability

The original contributions presented in this study are included in the article. Further inquiries can be directed to the corresponding authors.

## Supplementary Materials

The following supporting information can be downloaded at: https://www.mdpi.com/article/10.3390/nano15231820/s1, Figure S1: The SEM photographs of (a,b) H-SAPO-35 and (c,d) Na-SAPO-35; Figure S2: (a) The formation of Bronsted acids in molecular sieves. (b) The formation mechanism of π-complex and carbocation C_3_H_7_^+^. (c) The formation mechanism of oligomer; Table S1: Textural properties of samples; Table S2: The molar composition of samples; Table S3: C_3_H_6_ and C_3_H_8_ uptakes as well as C_3_H_6_/C_3_H_8_ selectivities for state-of-the-art zeolite-based adsorbents; Table S4: Dual-site Langmuir fitting parameters of propylene and propane adsorption isotherms on H-SAPO-35 and Na-SAPO-35; Table S5: Thermodynamic parameters of the adsorption of propylene and propane on H-SAPO-35 and Na-SAPO-35 molecular sieves. Refs. [44,45,46,47,48,49] are cited in the Supplementary Materials.
