# Supplementary material for: The Molecular Sieving of Propylene and Propane on SAPO-35 Molecular Sieve
Source: Nanomaterials (Basel). 2025 Dec 1;15(23):1820. doi: 10.3390/nano15231820 (PMC12693193; doi:10.3390/nano15231820)
Supplement: Supplementary file 1 [file nanomaterials-15-01820-s001.zip › nanomaterials-4003026-supplementary.pdf]

Article

The molecular sieving of propylene and propane on SAPO-35 molecular sieve

Yansi Tong [1,†], Kadi Hu [1,2,†], Qihao Yang [1,2,*], Hao Liu [1,2], Danhua Yuan [2,3], JunGang Wang [4], Mengting Lv [1], Hailong Wang [1], Ziqi Tian [1,2,*], Yunpeng Xu [2,3,*], and Liang Chen [1,2,*]

[1] Zhejiang Key Laboratory of Advanced Fuel Cells and Electrolyzers Technology, Ningbo Institute of Materials Technology and Engineering, Chinese Academy of Sciences, Ningbo 315201, China
[2] University of Chinese Academy of Sciences, Beijing 100049, China
[3] Dalian National Laboratory for Clean Energy, Dalian Institute of Chemical Physics, Chinese Academy of Sciences, Dalian 116023, China
[4] China State Key Laboratory of Coal Conversion, Institute of Coal Chemistry, Chinese Academy of Sciences, Taiyuan 030001, China
* Correspondence: yangqihao@nimte.ac.cn (Q.Y.); tianziqi@nimte.ac.cn (Z.T.); xuyunpeng@dicp.ac.cn (Y.X.); chenliang@nimte.ac.cn (L.C.)
† These authors contributed equally to this work.

Supplementary Note 1

When the adsorption isotherm can be well fitted by the traditional Langmuir model (Equation 1), Henry law constant was calculated by Equation 2[44].

$$q = q_{\mathrm{m}} \frac{bP}{1+bP} \quad (1)$$

$$K_H = q_{\mathrm{m}} b \quad (2)$$

When the adsorption isotherm must be fitted with a more complicated model, then Henry law constant could be obtained on the base of virial form of the thermodynamic equilibrium relation, as shown in Equation 3[45].

$$P = \frac{q}{K_H} \exp\left(2A_1 q + \frac{3}{2} A_2 q^2 + \frac{4}{3} A_3 q^3 + \cdots\right) \quad (3)$$

Where A_i represents the virial coefficient, (i=1, 2, 3, …). When the adsorption amount (q) is very low, higher order terms can be ignored, so the relation between ln(P/q) and q should approach linearity, and the intercept of this line is $-K_H$.

$$\ln\left(\frac{P}{q}\right) = A_1 - \ln K_H \quad (4)$$

The Henry constant can be extracted by extrapolating the intercept, and the temperature-dependent Henry constants were used to calculate the heat of adsorption at zero-coverage by the following van't Hoff equation[44]:

$$K_H = K_{H0} \exp\left(\frac{-\Delta U^0}{RT}\right) \quad (5)$$

Where ΔU^0 (J mol^{-1}) represents the adsorption heat at zero coverage,K_{H0} (mol kg^{-1} Pa^{-1}) represents the van't Hoff coefficient, R (J mol^{-1} K^{-1}) represents the universal gas constant, T (K) represents the temperature. So ΔU^0 could be obtained by establishing the relation between $\ln K_H$ and $1/T$.

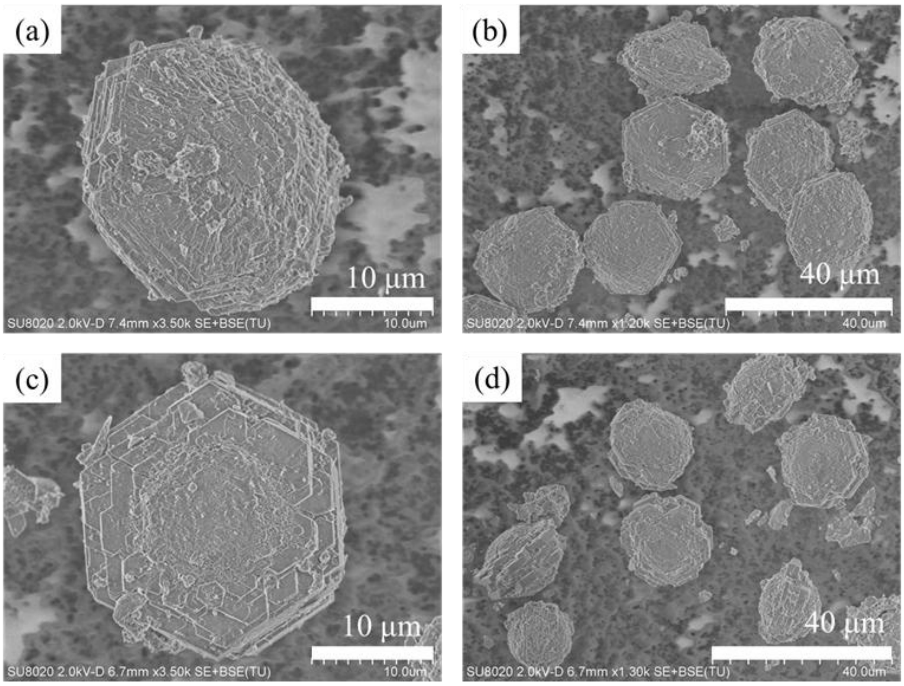

Supplementary Figure S1. The SEM photographs of (a-b) H-SAPO-35 and (c-d) Na-SAPO-35.

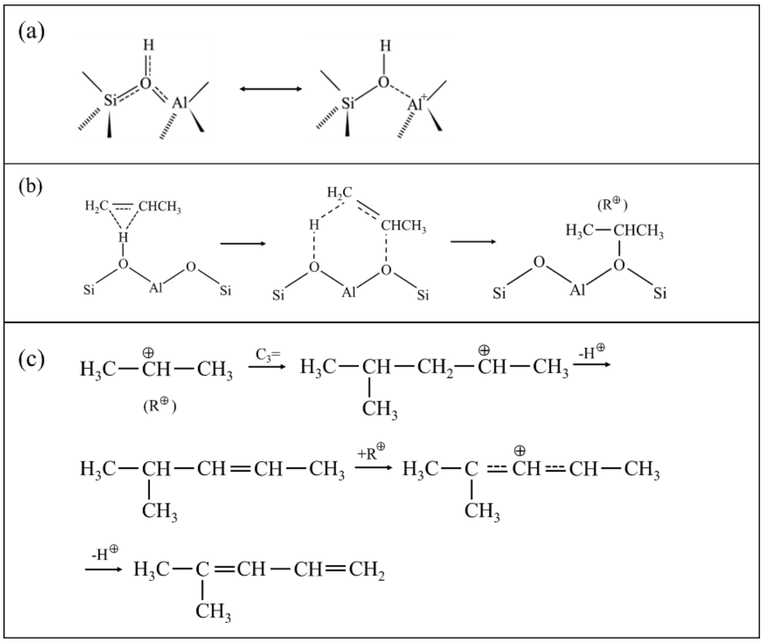

Supplementary Figure S2. (a) The formation of Bronsted acids in molecular sieves. (b) The formation mechanism of π-complex and carbocation $C_3H_7^+$. (c) The formation mechanism of oligomer.

Supplementary Table S1. Textural properties of samples

Samples	Surface area (m^2 g^{-1})			Pore volume (cm^3 g^{-1})	
	S_{total}[a]	S_{micro}[b]	S_{ext}[c]	V_{total}[d]	V_{micro}[e]
H-SAPO-35	375	345	30	0.21	0.17
Na-SAPO-35	299	255	44	0.17	0.13
Regenerated Na-SAPO-35	298	252	46	0.17	0.13

[a] S_{total} represents BET surface area, [b] S_{micro} represents t-Plot micropore area, [c] S_{ext} represents external surface area, [d] V_{total} represents total pore volume, [e] V_{micro} represents t-Plot micropore volume.

Supplementary Table S2. The molar composition of samples

Samples	Molar composition
H-SAPO-35	$Al_{0.505}Si_{0.028}P_{0.467}O_2$
Na-SAPO-35	$Na_{0.027}Al_{0.512}Si_{0.027}P_{0.461}O_2$

Supplementary Table S3. C_3H_6 and C_3H_8 uptakes as well as C_3H_6/C_3H_8 selectivities for state-of-the-art zeolite-based adsorbents

Materials	C_3H_6 uptake (mmol g^{-1})	C_3H_8 uptake (mmol g^{-1})	Selectivity
CaA[33]	2.6	2.2	1.2
NaA[33]	1.8	0.3	6.7
ITQ-12[46]	1.2	0.5	2.4
ITQ-29[47]	2.4	2.1	1.2
ITQ-32[48]	1.2	0.4	3.0
DD3R[29]	1.5	0.8	1.9
Si-CHA[49]	2.8	1.9	1.5
AlPO-14[9]	0.7	0.06	12
Na-SAPO-35	1.14	0.1	11.4

Supplementary Table S4. Dual-site Langmuir fitting parameters of propylene and propane adsorption isotherms on H-SAPO-35 and Na-SAPO-35

Samples	Adsorbates	q_A	b_A	q_B	b_B	R^2
H-SAPO-35	Propylene	1.59	3.30	1.91	1.17×10^{-3}	0.9999
	Propane	0.62	0.85	0.35	1.01×10^{-2}	0.9987
Na-SAPO-35	Propylene	1.05	3.08	7.61	1.29×10^{-4}	0.9993
	Propane	0.03	0.31	0.16	7.42×10^{-3}	0.9999

Supplementary Table S5. Thermodynamic parameters of the adsorption of propylene and propane on H-SAPO-35 and Na-SAPO-35 molecular sieves

Samples	Adsorbates	K_H, 298 K (mol kg^{-1} Pa^{-1})	Adsorption heat (kJ mol^{-1})	$K_H(C_3H_6)$/ $K_H(C_3H_8)$
H-SAPO-35	Propylene	4.04×10^{-3}	22.8	7.5
	Propane	5.37×10^{-4}	-	
Na-SAPO-35	Propylene	2.86×10^{-3}	17.6	255
	Propane	1.12×10^{-5}	-	
